# Tanzania national survey on iodine deficiency: impact after twelve years of salt iodation

**DOI:** 10.1186/1471-2458-9-319

**Published:** 2009-09-03

**Authors:** Vincent D Assey, Stefan Peterson, Sabas Kimboka, Daniel Ngemera, Celestin Mgoba, Deusdedit M Ruhiye, Godwin D Ndossi, Ted Greiner, Thorkild Tylleskär

**Affiliations:** 1 Tanzania Food and Nutrition Centre, 22 Ocean Road, P. O. Box 977 Dar es Salaam, Tanzania; 2 National Council for Prevention and Control of Iodine Deficiency Disorders(NCCIDD) P.O. Box 977 Dar Es Salaam,Tanzania; 3 Centre for International Health, University of Bergen, Årstadveien 21, N-5009 Bergen, Norway; 4 Department of Women's and Children's Health, Unit of International Maternal and Child Health (IMCH), *Akademiska Sjukhuset*, Uppsala University, SE -75185 Uppsala, Sweden; 5 Department of Public Health Sciences, Division of International Health (IHCAR), Nobel v 9. Karolinska Institutet S-17177 Stockholm, Sweden; 6 UNICEF Kitgum Zonal Office, c/o UNICEF Kampala, Plot 9, George Street, P.O. Box 7047 Kampala Uganda; 7 Food and Nutrition Department, Hanyang University, 17 Haengdang-dong, Seongdong-gu Seoul 133-790, South Korea

## Abstract

**Background:**

In many low-income countries, children are at high risk of iodine deficiency disorders, including brain damage. In the early 1990s, Tanzania, a country that previously suffered from moderate to severe iodine deficiency, adopted universal salt iodation (USI) as an intervention strategy, but its impact remained unknown.

**Methods:**

We report on the first national survey in mainland Tanzania, conducted in 2004 to assess the extent to which iodated salt was used and its apparent impact on the total goitre prevalence (TGP) and urinary iodine concentrations (UIC) among the schoolchildren after USI was initiated. In 2004, a cross-sectional goitre survey was conducted; covering 140,758 schoolchildren aged 6 - 18 years were graded for goitre according to new WHO goitre classification system. Comparisons were made with district surveys conducted throughout most of the country during the 1980s and 90s. 131,941 salt samples from households were tested for iodine using rapid field test kits. UIC was determined spectrophotometrically using the ammonium persulfate digestion method in 4523 sub-sampled children.

**Results:**

83.6% (95% CI: 83.4 - 83.8) of salt samples tested positive for iodine. Whereas the TGP was about 25% on average in the earlier surveys, it was 6.9% (95%CI: 6.8-7.0) in 2004. The TGP for the younger children, 6-9 years old, was 4.2% (95%CI: 4.0-4.4), n = 41,965. In the 27 goitre-endemic districts, TGP decreased from 61% (1980s) to 12.3% (2004). The median UIC was 204 (95% CF: 192-215) μg/L. Only 25% of children had UIC <100 μg/L and 35% were ≥ 300 μg/L, indicating low and excess iodine intake, respectively.

**Conclusion:**

Our study demonstrates a marked improvement in iodine nutrition in Tanzania, twelve years after the initiation of salt iodation programme. The challenge in sustaining IDD elimination in Tanzania is now two-fold: to better reach the areas with low coverage of iodated salt, and to reduce iodine intake in areas where it is excessive. Particular attention is needed in improving quality control at production level and perhaps the national salt iodation regulations may need to be reviewed.

## Background

Iodine is required for the production of thyroid hormones, which are essential for normal brain development [1]. Lack of iodine at conception causes maternal hypothyroidism, which has dramatic consequences for the foetus, leading to severe and irreversible brain damage. These consequences can be prevented by the correction of iodine deficiency (ID) before pregnancy [2] through iodated salt (I-salt) or iodine supplements to pregnant and lactating women [3]. It is estimated that two billion people, or 30.6% of the global population, have insufficient iodine intake, including 59.7 million school-age children in Africa, out of which 21.9 million are in the Eastern and Southern African region [4,5].

In mainland Tanzania, 41% of the population live in geographic areas subjected to iodine-deficiency [6], and many scattered surveys conducted around the country during the 1980s showed that an estimated 25% suffered from some form of iodine deficiency disorders (IDD) [7]. In some areas in the Southern Highlands, the total goitre prevalence (TGP) reached as high as 90% and half the school children were hypothyroid [8]. These areas were among the 27 districts targeted for iodized oil capsules, which were provided biannually to everyone 1-45 years old from 1985 [9]. As universal salt iodation (USI) was satisfactorily implemented, gradually the capsules were phased out in 1996 [10].

USI was achieved through a public-private partnership formed in 1985 under the coordination of Tanzania's National Council for Control of IDD (NCCIDD) [9,11]. However, Tanzania has a multi-faceted salt sector, with more than 6500 salt producers dispersed over the entire country. There is a high variation in scales and in quality of salt production and in iodation technology [12]. Thus sustainable elimination of IDD, achievement of which was originally hoped for by the end of the year 2005, still remains a challenge. In an effort to streamline I-salt production and distribution, the Tanzania Salt Producers Association (TASPA) was formed in 1994 [13]. It is currently playing a pivotal role in unifying salt producers countrywide, especially small-scale producers, easing the provision of technical support and the monitoring of USI.

Spot surveys conducted in the high and low IDD endemic areas in 1999 and 2001 showed great variability in both the process (availability of iodated salt) and impact indicators for iodine deficiency (ID) [14,15]. This paper presents the results from the first national survey in mainland Tanzania, conducted in 2004 to assess the extent of iodated salt use, to measure urinary iodine concentrations (UIC), and to estimate TGP among schoolchildren approximately twelve years after salt iodation was initiated in Tanzania.

## Methods

### Study area, design and sampling

**Figure 1:**
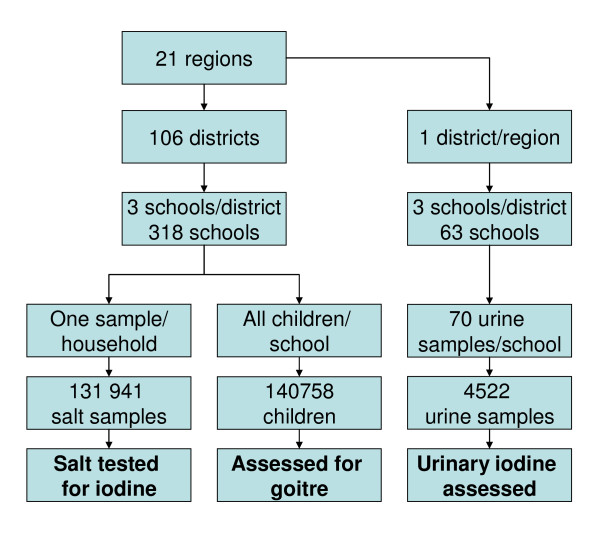
**Summary of the survey sampling procedure**.

In 2004, Tanzania had an estimated mainland population of 35.7 million projected from 2002 census housing population [16], divided into 21 administrative regions and 106 districts each of which was sampled in this reported cross-sectional national survey. Surveyed districts were regarded as clusters at regional level. The survey took place among school-age children, considered to be an appropriate study group for IDD surveys [1]. Three schools each from one of the three strata known to influence ID were selected in each district: one township and two rural, i.e., one from a high and one from a low altitude (Figure 1). Each school was randomly selected from a list of schools in each stratum. This approach was identical to the one used in the 1980s' surveys, in order to increase the chances of our results being comparable to those [6]. The schools were summed to reflect the number of surveyed children per region. The study included a total of 131,941 household salt sample tests for iodine and 140,758 school children aged 6 - 18 years for goitre palpation, of whom 94,046 were 6 - 12 years old (Additional file 1 and 2). Normally, primary schools in Tanzania consist of classes one to seven. A sub-sample of systematically sampled 4523 school children palpated for goitre in 63 of the 318 schools gave urine for determination of UIC, Additonal File2, of which 2640 were in the age-group of 6-12 years, Additional file 1.

Tanzania Food and Nutrition Centre (TFNC) staff with relevant research experience [17] prepared the survey protocol. Each region was requested to nominate one competent medical officer to be the regional principal investigator (RPIs). All 21 RPIs were trained in survey procedures and protocol by TFNC staff, emphasizing the standardized assessment of goitre by palpation [17]. However, intra- and inter- observer variation for palpation of goitre was not formally assessed. The RPIs later on trained district staff to assist them in collecting the non-palpation survey data, while the RPIs carried out all the goitre palpation and TFNC staff (some co-authors SK, DMR & VDA) supervised the execution of the study in each region.

### Coverage of iodated salt at household level

From each household with children attending a sampled school, one child was requested to bring to school a teaspoonful of the family's salt wrapped in paper. A rapid field test kit (MBI KITS, Madras, India), were used to determine the percentage of salt samples with iodine concentration above and below 15 ppm (mg/kg of salt) [18,19]. Precautions were taken to ensure the viability of the test kits by testing 125 kits randomly chosen from the batch of 30 gross boxes of MBI KITS used for the survey. None of kits were defective in identifying salt known to be either iodated or non-iodated. Furthermore, each RPI was given iodated salt prepared at TFNC as control material to countercheck the kits before testing household salt. This may also have reduced the multiple-observer errors that have been found elsewhere using MBI kits [19]. Districts were categorized as "adequate" (>90%) [1], unsatisfactory (76 - 90%), poor (50 - 75%), or very poor (<50%) in terms of the percentage of households using iodated salt.

### Goitre prevalence

In each sampled school, all pupils (6 - 18 years old) who attended on the day of the survey were examined and graded according to the current WHO goitre classification system. Each subject was categorized as goitrous if the thyroid size was of grade 1 (i.e., goitre is palpable but not visible in normal position) or grade 2 (i.e. a swelling in the neck that is clearly visible and is consistent with an enlarged thyroid when the neck is palpated)[1]. TGP is referred as a sum of goitre grade 1 and 2 (visible). The regional TGP was calculated for children 6 - 12 years old as proposed by WHO [1] and for the younger children 6 - 9 years old. TGP from 0 - 4.9% was not considered of public health significance, while areas with 5 - 19.9%, 20 - 29.9% and ≥30% were ranked as having mild, moderate and severe IDD, respectively. To compare the 2004 regional goitre prevalences to those obtained in the 1980s, we also estimated TGP for children aged 6 - 18 years (Additional file 2).

### Urinary iodine concentration

Spot urine samples were collected in one district randomly selected from each region's listed districts. In each of the three schools previously selected for goitre palpation, ten children (equal numbers of boys and girls) were selected from each class by systematic sampling. UIC was used as a proxy indicator of iodine status at population level [1].

Each sampled child provided 10 - 15 ml urine in a tightly closed, clean 20 ml glass bottle. These urine samples were analyzed using the ammonium persulfate digestion method, based on the Sandell-Kolthoff reaction [20], at the National Resource Laboratory for Iodine located at TFNC, which also supports IDD programmes in the Eastern Africa region (certified as "satisfactory participant" in the program for ensuring quality of urinary iodine procedures (EQUIP)). The accuracy of the assay was assessed using reference quality-control urine specimens supplied by Centres for Disease Control and Prevention (CDC), Atlanta, Georgia, USA; the detection limit was <5.0 μg/L. The coefficient of variation of this assay in our laboratory was 10%, which concurred to other findings from a reference method [21].

According to WHO [1], UIC of school children should be used to categorized the severity of ID as follows: 0 - 19.9 and 20 - 49.9 μg/L for very insufficient/insufficient, indicating severe and moderate, respectively; 50 - 99.9 μg/L for mildly insufficient; 100 - 199.9 for 'optimal'; 200 - 299.9 μg/L for 'above requirements'; and above 300 μg/L for 'excessive' intake. The median UIC should be ≥100 μg/L in school-age children, with no more than 50% of urine samples <100 μg/L and no more than 20% <50 μg/L [1].

### Data processing and analysis

Data were keyed into Excel 2003 at TFNC, entries were validated through frequency and cross-tabulations, and errors rectified against original data forms. In one region the goitre prevalence still seemed unexpectedly high and the goitre examination was repeated by TFNC staff.

Data were analyzed using Statistical Package of Social Sciences (SPSS) version 11.0. The Mann-Whitney *U*-test was used to compare the sexes, age-groups, areas and time intervals for the estimated TGP in the 1980s and 2004 and the I-salt coverage in the ID areas in 1999 and 2004. An Eta squared calculation was used to measure the intervention effect on TGP and to show the difference among groups before and after intervention. The confidence intervals for the median were calculated according to Gardner & Altman [22].

To visualise the severity of the iodine deficiency, traffic light colours (colour coded system) were used for simple and quick interpretation and understanding of the findings. For I-salt coverage a green (G) means a coverage above 90% (adequate); yellow (Y) means 50 - 90% (poor/unsatisfactory) and red (R) means 0 - 49.9% (very poor). For TGP a green means 0 - 4.9% (not of public health significance); yellow (Y) means 5 - 19.9% (mild); orange (O) means 20 - 29.9% (moderate) and red (R) means a total goitre prevalence above 30% (severe). For median urinary iodine red (R) means a median of 0 - 49.9 μg/L (very insufficient); yellow (Y) means 50 - 99.9 μg/L (insufficient); green (G) means 100 - 299.9 μg/L (optimal and above requirement) and purple (P) means a median above 300 μg/L (excessive intake).

### Ethical approval

This study proposal was approved by TFNC's Committee on Research on Human Subjects, which did not require parental consent for each school child. The RPIs communicated in advance to the district and community leaders, primary school teachers and the schoolchildren, explaining the purpose and importance of the study. Oral assent was obtained from participating school children; none refused to participate.

## Results

### Availability of I-salt

Of the 131,941 salt samples tested, 83.6% (95% confidence interval (95% CI): 83.4 - 83.8%) contained iodine. The regional proportions of salt samples with iodine ranged from 25.4% in Lindi to 99.7% in Kagera (Additional file 1).

**Figure 2:**
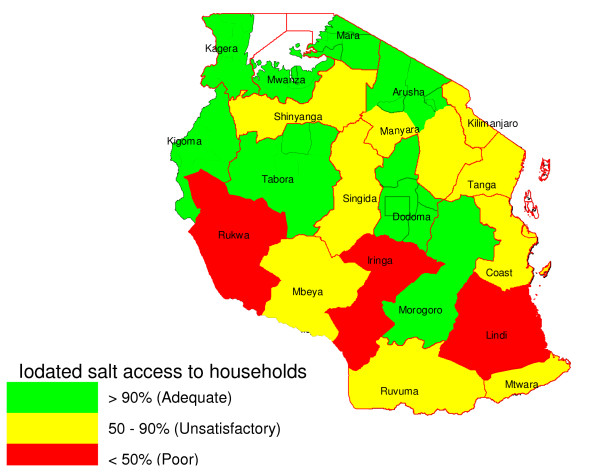
**Iodated salt coverage at household level by region in 2004**.

The availability of I-salt was very poor (<50%) in three of the 21 regions, Lindi, Iringa and Rukwa. 58 of the 106 districts (55%) had >90% I-salt coverage while 16, 18 and 14 districts had unsatisfactory (76 - 90%), poor (50 - 75%) and very poor (<50%) coverage at household level, respectively. In the urban areas, 88% of households had I-salt compared to 81% in rural areas, *p < 0.001*. In the districts sampled for UIC determination (Additional file 1), 85.3% (95% CI: 84.9 - 85.8%) had I-salt suggesting that they were a relatively un-biased sub-sample. Regions along the coast of the Indian Ocean and others that had small-scale salt-production (often without iodation) had unsatisfactory to poor I-salt coverage (Figure 2).

### Goitre prevalence

**Figure 3:**
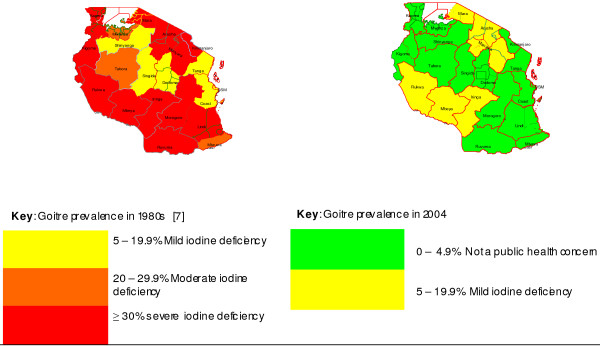
**Total goitre prevalence by region before and after USI intervention in Tanzania**.

The overall regional TGP at 6 - 12 years of age was 5.5% (95% CI: 5.3 - 5.6). Only six regions had a TGP indicating mild ID (5.0 - 19.9%), while the rest had less than 5% (Additional file 1 & Figure 3). Among the youngest children 6 - 9 years old, the TGP was 4.2% (95% CI: 4.0 - 4.4), n = 41,965. The TGP in the 21 districts selected for urinary iodine determination at 6 - 12 years of age was 5.4% (95% CI 5.3 - 5.5%), again identical to the total regional goitre prevalence (Additional file 1).

Among all school children aged 6 - 18 years, the TGP was 6.9% (95% CI: 6.8 - 7.1%) (Additional file 2) and the prevalence of visible goitre was only 0.3%. Mild (TGP 5.0 - 19.9%) and moderate (20 - 29.9%) IDD was seen in two and in eight regions. Rural areas had a higher goitre prevalence (8.0%) than urban areas (5.5%), *p < 0.001*. Goitre was found in 6.5% of boys and 7.4% of girls, *p < 0.001*. The overall distribution of TGP increased with age, *p < 0.05*, with the highest prevalence observed in those over 16 years; see Table 1. Two districts, Mufindi in Iringa and Mbarali in Mbeya regions had a TGP of 31.3% and 38.9%, respectively, indicating severe IDD. However, no district had a TGP indicative of severe IDD in the age-group 6 - 12 years.

**Table 1:** Distribution of total goitre prevalence and median urinary iodine concentration by age group

**Age-group (years)**	**Total goiter prevalence**			**Urinary iodine concentration**	
	
	**Total children surveyed (N)**	**Children with goitre (n)**	**% children with goitre (95% CI)**	**Urine samples analysed (N)**	**Median UIC μg/L (95% CI)**
6 -12	94046	5181	5.5 (5.3, 5.6)	2640	203.3 (187, 219)
13 - 15	40533	3881	9.6 (9.3, 9.9)	1555	210.0 (192, 228)
16 and above	6179	707	11.4 (10.6, 12.2)	328	185.6 (138, 233)

Total	140758	9769	6.9 (6.8, 7.1)	4523	203.6 (192, 215)

Confidence intervals of the median constructed by the method described by Gardner and Altman [22].

The data in Table 2 show that TGP in the 27 originally IDD-endemic districts appear to have decreased from an unweighted average of 60.7% in the 1980s to 12.3% in 2004, (9.0% at age 6 - 12 years). The calculated Eta squared statistics was 0.90 (t Stat = 15.4, n = 27) indicating a large effect size for this decline.

**Table 2:** Change in total goitre prevalence before* and after** introduction of salt iodation in the 27 originally most iodine deficient districts in Tanzania

**Region**	**District**	**Total goitre** **1980s (%)** 6 - 18 years*	**Traffic light status**	**Total goitre** **2004 (%)** 6 - 18 years**	**Traffic light status**	**Total goitre change in % age points by 2004**	**Total goitre** **2004 (%)** **6 - 12 years**	**Traffic light status**
Mbeya	Mbeya	88.0	R	24.2	O	-63.8	19.5	Y
Mbeya	Ileje	86.0	R	15.0	Y	-71.0	11.7	Y
Mbeya	Mbozi	83.0	R	21.0	O	-62.0	17.3	Y
Rukwa	Nkasi	81.0	R	24.9	O	-56.1	17.3	Y
Iringa	Mufindi	80.9	R	31.3	R	-49.6	27.1	O
Morogoro	Ulanga	79.0	R	9.1	Y	-69.9	7.8	Y
Rukwa	Sumbawanga	79.0	R	19.8	Y	-59.2	11.5	Y
Mbeya	Kyela	78.6	R	19.3	Y	-59.3	18.5	Y
Ruvuma	Songea	75.0	R	6.9	Y	-68.1	1.9	G
Rukwa	Mpanda	70.0	R	17.9	Y	-52.1	12.5	Y
Mbeya	Rungwe	68.0	R	19.2	Y	-48.8	15.9	Y
Kagera	Ngara	67.7	R	1.7	G	-66.0	0.8	G
Kigoma	Kasulu	64.0	R	3.8	G	-60.2	2.9	G
Dodoma	Mpwapwa	59.0	R	3.2	G	-55.8	1.6	G
Kigoma	Kibondo	58.2	R	2.7	G	-55.5	5.0	Y
Kilimanjaro	Rombo	54.0	R	3.8	G	-50.2	2.2	G
Kigoma	Kigoma	54.0	R	4.8	G	-49.2	1.4	G
Arusha	Monduli	54.0	R	17.0	Y	-37.0	16.3	Y
Iringa	Makete	49.9	R	18.8	Y	-31.1	13.1	Y
Iringa	Njombe	49.9	R	24.9	O	-25.0	18.0	Y
Mbeya	Chunya	49.2	R	0.0	G	-49.2	0.0	G
Arusha	Arumeru	45.4	R	11.4	Y	-34.0	11.3	Y
Kagera	Biharamulo	42.3	R	1.2	G	-41.1	0.6	G
Ruvuma	Mbinga	36.3	R	5.5	G	-30.8	0.7	G
Iringa	Ludewa	31.3	R	19.1	Y	-12.2	15.9	Y
Kagera	Karagwe	31.2	R	2.7	G	-28.5	1.1	G
Kagera	Bukoba	23.4	O	1.9	G	-21.5	1.1	G

**Total**		**60.7**	**R**	**12.3**	**Y**	**-48.4**	**9.0**	**Y**

Source: [6] & Bunga *et al *1991 TFNC Report No. 1429 An evaluation of the impact of the distributed IOC in Tanzania (Unpublished).

Mann-Whitney *U *test or paired t-test and Eta squared statistics were used to calculate the magnitude of the intervention's effect. Eta = 0.90, t = 15.43, p < .0001 (two-tailed), 95% CI: 15.43 ± 2.06, n = 27.

Key traffic light alphabetical colour codes: Total goitre prevalence: 0 - 4.9% (not of public health significance) = green (G), 5 - 19.9% (mild) = yellow (Y), 20 - 29.9% (moderate) = orange (O), ≥ 30% (severe) = red (R),

### Urinary iodine concentration

The overall country-wide median UIC for 6 - 12 years and for 6 - 18 years was 203 and 204 μg/L, respectively (Additional file 1and Additional file 2), while this falls within the ID status category of "over iodine requirements" i.e., >200 and <300 μg/L, the 95% confidence interval includes the optimal range of 100-200 μg/L. The regional/district medians ranged from 45.1 μg/L - 887 μg/L (Additional file 2). The median UICs in boys and girls were 205 μg/L (n = 2265) and 204 μg/L (n = 2258), respectively; indicating no sex difference *(p = 0.973)*. Similarly there was no age difference in UIC; median UIC value for the eldest age group 13 -18 years was 204 (95% CI: 187- 221) μg/L (Table 1), compared to 206 (95% CI: 184 - 227) μg/L, n = 1264) for the youngest children 6 - 9 years.

Five districts (Additional file 1) had median UICs below 100 μg/L and the highest proportion of individuals with UIC values below 100 μg/L (range 54 - 83%), all above the WHO cut-off of 50%; Namtumbo district in Ruvuma region had the lowest median (45 μg/L). Five other districts had excessive median UIC values ≥ 300 μg/L. The median UIC in the urban population was higher (250 μg/L) than in the rural population (182 μg/L), *p = 0.0001*. The overall proportions of individual UICs under 100 μg/L and under 50 μg/L were below the WHO thresholds of 50% and 20%, respectively, for low iodine intake. Only 24% fell within the range of optimal iodine intake (100 - 199.9 μg/L) while 35.2% had an iodine intake exceeding ≥ 300 μg/L (Table 3). The situation was similar when examined in the age-group 6-12 years (Additional file 1) and in all age-group 6-18 years (Additional file 2). The higher the median UIC level, the high the proportion of individuals with UIC ≥ 300 μg/L and vice versa was also true (Additional file 1 & Additional file 2).

**Table 3:** Proportion of children in each WHO urinary iodine category [1]

**Population's status of iodine intake**	**Urinary iodine levels (μg/L)**	**Proportion of children**		
		
		**n**	**%**	**Cumulative %**
Very insufficient (severe iodine deficiency)	Below 20	92	2.0	2.0
Insufficient (moderate iodine deficiency)	20 - 49.9	382	8.5	10.5
Insufficient (mild iodine deficiency)	50 - 99.9	654	14.5	25.0
Adequate iodine nutrition	100 - 199.9	1109	24.5	49.5
Above requirements (poses a slight risk*)	200 - 299.9	692	15.3	64.8
Excessive intake (poses a clear risk*)	300 and above	1594	35.2	100.0

**Total**		**4523**	**100.0**	

*Risk of adverse health consequences (iodine-induced hyperthyroidism and autoimmune thyroid diseases)

## Discussion

In 2003, WHO estimated that 45% of the population of Eastern Africa was iodine insufficient [23]. The present study demonstrated an impressive improvement in iodine nutrition in Tanzania 12 years after the initiation of the USI. Tanzania has moved from a situation where an estimated 25% of its population was vulnerable to iodine deficiency [9] to one where 84% consume I-salt and 94.5% of the 6 - 12 year olds have normal sized thyroid glands [1]. In spite of an adequate median iodine intake at the national level, this survey revealed large regional variation with evidence of iodine deficiency in some areas and of excessive intakes in other areas.

### Availability of I-salt

Out of the three indicators - percentage of households with access to I-salt, TGP and UIC, concordance was observed for I-salt and UIC while TGP was inversely but less strongly correlated with the I-salt and UIC increase. When more than 90% of households in a region/district are covered by I-salt, the UIC was likely to be adequate (or high), while TGP was low and vice versa [1,3]. There are some exceptions; for example, Lindi and Mtwara regions reported low I-salt coverage and low UIC but very low goitre prevalence. There are other areas where concordance is poor in the other direction, for example Mbeya where I salt, UIC and TGP are all high while in Rukwa region there was low I-salt with high UIC and TGP. But, as shown in Table 3, all these regions of exceptionally high TGP in the 80s and have shown dramatic improvement. Presumably the process of bringing down TGP rates simply takes more time in such settings [1,24].

In many areas weaknesses in salt quality assurance in salt production that include inconsistencies in salt/iodate mixing, consumer's preference for uncontrolled backyard salt production, and non-adherence to salt regulations by salt producers and traders are reflected in low coverage of I-salt and in the high or low levels of iodine [11,25]. The coverage of I-salt at household level obtained using test kit method in this study and in our previous study [14] showed its concordance with UIC. The UIC data suggest that, despite some concerns about the reliability of the kits, they can be a useful indicator for program monitoring, allowing for large samples to be assessed at a reasonable cost [19]. However, the kit can be used only to detect the presence or absence of iodine, not amounts added.

### Reasons for iodine variations

Lindi, Mtwara and Iringa (regions with a proliferation of small-scale salt producers) showed poor coverage of I-salt and a high proportion of individuals with low UIC levels (above the 50% threshold), indicating inadequate dietary iodine intake. Although the TGP for these regions (all of which, except Iringa, are situated along the coast of Indian Ocean) was low, IDD, including brain damage, can occur unless iodine enters the food chain through addition of iodine to foods [2,26].

The difficulty of providing a continuous supply of potassium iodate and the poor iodation technologies in many small-scale salt producers could be factors in the shortage of I-salt in households and the iodine variability observed in this survey [14,27]. Increased use of appropriate technology from recently modified iodation procedures using local spray pumps with manual mixing under close supervision [25], and the creation of a system for consumers to contribute to the costs of potassium iodate may reverse the situation and sustain salt iodation in Tanzania.

### Goitre prevalence

Goitre prevalence responds slowly to intervention, lagging 6 months to several years behind increased iodine intake, depending on many factors [1]. Nevertheless, goitre was a useful indicator for the 6 - 12 age-group in our study, given that iodized oil capsule distribution started in 1986 (gradually being phased out by 2000) and salt iodation began about 12 years ago.

Fifteen (71%) of the 21 regions now have a TGP of non-public health significance [1]. The remaining regions have mild to moderate IDD. Compared to past survey results, the TGP among 6 - 18 year olds has decreased substantially; cases of visible goitre are now negligible (0.3%) compared to an unweighted mean of 11% in the 1980s. This suggests that Tanzania's two decades of effort to combat IDD have had a marked impact [1,9]. The TGP in the lower age group (6 - 9 years) with median UIC within the "above requirements" category [1], suggests that iodine intake is adequate - a sign that IDD is almost completely eliminated in this group. This agrees with other findings elsewhere that once USI has picked up well, younger age-groups will have TGP less than 5%, while in the older age will remain above that level for some years after reaching adequate iodine nutrition [28].

The higher goitre prevalence (>9.6%) seen among 13 - 18 year olds may not reflect the true IDD situation [1] but could rather be ascribed to slow regression of goitres from early childhood ID [1]. It could also be the effect of the increased metabolic iodine demand in juveniles, which tends to cause thyroid gland enlargement [24]. TGP in the previously most goitre-affected areas decreased by over 50 percentage points between 1980s and 2004, indicating a very large effect size of the USI intervention (Eta = 0.90). However, the country still has pockets of moderate to mild ID, which requires further action since the goal of USI is not simply to increase UIC but to eliminate thyroid dysfunction caused by ID [28]. Tanzania's IDD situation requires more attention in ensuring balance of optimal iodine nutrition. As has been previously emphasized, surveys should be conducted more frequently in high IDD risk areas [29].

The goitre palpation method is known to have low specificity and may not be useful for countries with successful USI programmes [17,30-32]. Thus, once the iodine status of population approaches normality as is now the case in Tanzania, ultrasound examination should be considered to demonstrate IDD elimination by the goitre criterion, and to validate other, more easily obtained indicators, like urinary iodine concentrations.

### Urinary iodine concentration

UIC has the obvious advantage that - unlike other indicators - it can sensitively detect excess iodine intake [1]. Its drawback is higher cost, limiting the number of samples that can be taken.

The overall median UIC falls within the category of "above requirements" [1], with 35% of urine samples suggesting excessive iodine intake (≥ 300 μg/L). Only about a quarter of the individual UICs were below 100 μg/L, which confirm that ID is not a significant public health problem [1]. The median UIC for the urban population was higher than for the rural, justifying the need to monitor iodine levels, especially in urban areas to prevent risk of disorders that may be associated with excessive intake [32,33].

### Reasons for excessive iodine intake

Excessive iodine intakes were observed among people in one or more of four scenarios: first was from those living close to salt factories (Temeke and Kisarawe districts), second scenario to those people living in commercial centres with access to I-salt direct from the factory that has not passed through the steps in the salt marketing chain known to cause iodine loss [9,34] (Temeke and Kisarawe districts in the Dar es Salaam area), and third was in those people living in the Mwanza area, where dried fish products are commonly consumed from Lake Victoria [35] and preservation was usually with I-salt (Ilemela district). However, despite of the fact that districts with high access to marine/lake seaweeds and fish may have high iodine intake, earlier research in Tanzania has reported inadequate iodine status in other areas with easy access to these foods [15,35,36].

The fourth scenario was observed from population living close to Mombasa, Kenya (which started to iodate salt earlier than Tanzania and at the same high iodine level) [37] where, even more so than in Tanzania, improved distribution, packaging and handling of I-salt, which has probably reduced iodine losses, making such high iodine content at factory levels inappropriate[11] (Hai and Simanjiro districts).

Other countries in the Eastern Africa region have reported a similar trend of excessive iodine intake (>30%) [12]. Inadequate quality control of iodation at salt producing factories may contribute to the problem [38]. It is likely that lower factory salt iodation levels, guidelines for which have recently been harmonized at 40 - 60 ppm in the Eastern and Southern Africa region, will reduce the prevalence of excessive iodine intake [12]. A sudden and large increase in iodine intake has been associated with increased risks of iodine-induced hyperthyroidism, sub clinical hypothyroidism, and autoimmune thyroiditis in individuals previously suffering from severe ID [32,33,39,40]. Most of these disorders are transient [26]. During the rolling out of the IOC campaigns and the introduction of USI, no clinical cases of iodine-induced hyperthyroidism were brought to the attention of IDD program staff in Tanzania [41]. Even if this might be due to incomplete surveillance, it is likely that the most critical period for iodine-induced hyperthyroidism is over after more than two decades of IDD program activity. Still, surveillance of iodine intake needs to continue in order to allow fine-tuning of the iodine concentration in the salt [1,39,42].

### Study limitations

This study documents a successful trend towards achieving and sustaining the elimination of IDD in mainland Tanzania. However, there were some limitations:

(i) To achieve comparability, sampling was both clustered (by district) and stratified by geographical location of schools for both I-salt and goitre palpation in an attempt to improve the comparability with 1980s surveys that used this procedure [6]. Only three schools were chosen per district, which means that statistical inference at district level is not possible. Further, the schools were not selected by a proportional-to-size method, nor were they a random sample at regional level. The number of districts per region ranged from 9 - 24 (Additional file 1), enough to secure an adequate sample size at that level. On the national level, the sample size was large and more likely to be reasonably representative. The urine sampling method involved collection in only one district per region. Since the TGP for these sub-sampled districts was nearly similar to the national level, it was probably not a biased sub-sample at that level.

(ii) Twenty-one carefully trained RPIs participated in palpating goitres, one for each region. We did not measure and cannot rule out inter- and intra-observer variations [1,17].

(ii) Quantitative (titrated) values for iodine content in salt from salt factories, shops and households were not included in this study. Nevertheless, very few salt samples from the households contained no iodine, indicating good progress in the USI programme. The extent of inter-observer (inter-rater) variations among the multiple-observers was not assessed; nor was the iodine results from MBI test kits validated using the titration method. Such data would have increased the validity of results obtained by this qualitative method as reported elsewhere [43]. Although it is known that MBI test kit results often overestimate the availability of iodated salt, it still remains an important tool for monitoring salt iodation programmes [19]. These earlier reported findings and precautions taken during the survey add strength to our findings, as does the consonance between the regional proportions of households with I-salt and UIC data.

Finally, further research on the adequacy of iodine status in vulnerable groups such as neonates and pregnant and lactating women is required before final conclusions are drawn about IDD elimination in Tanzania [3,44-46]. These groups were not included in our sample and they may have iodine deficiency, as was found to be the case in Mali, a country that started and successfully attained a similar I-salt coverage as Tanzania in the same year [47]. Other findings have also reported the adequate iodine intake in schoolchildren but not in pregnant and lactating women [43].

## Conclusion

Tanzania has moved from a situation where some 41% of her population lived under iodine-deficient conditions before the introduction of USI [7] to a situation where most of her population now consumes I-salt, making ID at most a mild problem. The challenge in sustaining IDD elimination in Tanzania is now two-fold: to better reach the areas with low coverage of I-salt, and to reduce iodine intake in areas where it is excessive.

## Competing interests

The authors declare that they have no competing interests.

## Authors' contributions

VDA, SP, SK, DMR contributed to the study protocol, design and execution of the survey. CM, VDA, SK, DN and TT contributed to data analysis. VDA, TG, SP, GDN, TT participated in interpreting the data, manuscript writing, revision, and in critical review of the article. All co-authors have seen and approved the final submitted manuscript version.

## Pre-publication history

The pre-publication history for this paper can be accessed here:



## Supplementary Material

Additional file 1 Proportion of use of iodated salt (I-salt)* at household level and TGP** at regional level and median UIC*** at district level in schoolchildren (6 – 12 years) in 2004 ordered according to regional I-salt coverage.
Click here for file

Additional file 2 Proportion of use of iodated salt (I-salt)* at household level, TGP** at regional level and median UIC*** at district level in schoolchildren (6 – 18 years) in 2004 ordered according to regional I-salt coverage.
Click here for file

## References

[B1] WHO/UNICEF/ICCIDD (2007). Assessment of iodine deficiency disorders and monitoring their elimination: A guide for programme managers.

[B2] Hetzel BS (2005). Towards the global elimination of brain damage due to iodine deficiency: The role of the International Council for Control of Iodine Deficiency Disorders. International Journal of Epidemiology.

[B3] WHO/UNICEF (2007). Joint Statement by the World Health Organization and the United Nations Children's Fund.

[B4] UNICEF (2007). Iodine deficiency: a continuing threat to development. How seven countries in Eastern and Southern Africa are meeting this threat.

[B5] de Benoist B, McLean E, Andersson M, Rogers L (2008). Iodine deficiency in 2007: Global progress made since 2003. Food and Nutrition Bulletin.

[B6] Kavishe F, Maletnlema T, van de Haar F, Kavishe F (1987). Iodine deficiency disorders in Tanzania. Iodine deficiency disorders in the region Eastern, Central and Southern Africa Volume NINI/ICFSN public No5.

[B7] Haar F van der, Kavishe P, Medhin M (1988). The public health importance of IDD in Tanzania. Centr Afr J Med.

[B8] Wachter W, Mvungi M, Konig A, Pickardt C, Scriba P (1986). Prevalence of goitre and hypothyroidism in Southern Tanzania: effect of iodised oil on thyroid hormone deficiency. J Epidemiol Community Health.

[B9] Kavishe F, Mushi S (1993). Nutrition Relevant Actions in Tanzania A case study for the XV Congress of the International Union of Nutrition Sciences.

[B10] Peterson S, Assey V, Forsberg BC, Greiner T, Kavishe FP, Mduma B, Rosling H, Sanga AB, Gebre-Medhin M (1999). Coverage and cost of iodized oil capsule distribution in Tanzania. Health Policy Plan.

[B11] Sullivan K, Houston R, Cervinskas J, Gorstein J, UNICEF/PAMM/MI/ICCIDD/WHO (1995). Monitoring universal salt iodization programs.

[B12] UNICEF (2007). Protecting Children's Brain Development: Strategic review on sustained universal salt iodization in Eastern and Southern Africa. Report of a workshop 25-26 April 2005.

[B13] Carlsson J, Ismail S, Jitta J, Tekle E (1999). Working with Nutrition: A comparative study of Tanzania Food and Nutrition Centre and the National Nutrition Unit of Zimbabwe. Sida Evaluation 99/10.

[B14] Assey V, Mgoba C, Mlingi N, Sanga A, Ndossi G, Greiner T, Peterson S (2007). Remaining challenges in Tanzania's efforts to eliminate iodine deficiency. Public Health Nutrition.

[B15] Assey V, Greiner T, Mzee R, Abuu H, Mgoba C, Kimboka S, Peterson S (2006). Iodine deficiency persists in the Zanzibar Islands of Tanzania. Food Nutrition Bulletin.

[B16] Tanzania population and housing census. http://www.tanzania.go.tz/census/tanzaniatotal.htm.

[B17] Peterson S, Sanga A, Eklof H, Bunga B, Taube A, Gebre-Medhin M, Rosling H (2000). Classification of thyroid size by palpation and ultrasonography in field surveys. Lancet.

[B18] Diosady L, Mannar V, Geertman R (2000). Development of rapid test kits for monitoring salt iodization. 8th World symposium, conference.

[B19] Pandav C, Arora N, Krishnan A, Rajan Sankar R, Smita Pandav S, Karmarkar M (2000). Validation of spot-testing kits to determine iodine content in salt. Bulletin of the World Health Organization.

[B20] Pino S, Fang SL, Braverman LE (1996). Ammonium persulfate: a safe alternative oxidizing reagent for measuring urinary iodine. Clinical Chemistry.

[B21] Caldwell K, Makhmudov A, Jones R, Hollowell J (2005). EQUIP: a worldwide program to ensure the quality of urinary iodine procedures. Accreditation and Quality Assurance: Journal for Quality, Comparability and Reliability in Chemical Measurement.

[B22] Gardner M, Altman D (1989). Statistics with Confidence. British Medical Journal.

[B23] De Benoist B, Andersen M, Egli I, Takkouche B, Allen H, WHO (2004). Iodine Status Worldwide. WHO Global Database on Iodine Deficiency.

[B24] Aghini-Lombardi F, Antonangeli L, Pinchera A, Leoli F, Rago T, Bartolomei A, Vitti P (1997). Effect of iodized salt on thyroid volume of children living in an area previously characterized by moderate iodine deficiency. Journal of Clinical Endocrinology and Metabolism.

[B25] Assey V, Tylleskär T, Momburi P, Maganga M, Mlingi N, Reilly M, Greiner T, Peterson S (2009). Improved salt iodation methods for small-scale salt producers in low-resource settings in Tanzania. BMC Public Health.

[B26] Zimmermann M, Jooste P, Pandav C (2008). Iodine-deficiency disorders. The Lancet.

[B27] Assey VD, Peterson S, Greiner T (2008). Sustainable universal salt iodization in low-income countries - time to re-think strategies?. European Journal of Clinical Nutrition.

[B28] Zimmermann MB (2004). Assessing iodine status and monitoring progress of iodized salt programs. J Nutr.

[B29] Hetzel BS (1983). Iodine deficiency disorders (IDD) and their eradication. Lancet.

[B30] Chanoine JP, Toppet V, Lagasse R, Spehl M, Delange F (1991). Determination of thyroid volume by ultrasound from the neonatal period to late adolescence. Eur J Pediatr.

[B31] Delange F, Benker G, Caron P, Eber O, Ott W, Peter F, Podoba J, Simescu M, Szybinsky Z, Vertongen F (1997). Thyroid volume and urinary iodine in European schoolchildren: standardization of values for assessment of iodine deficiency. Eur J Endocrinol.

[B32] Zimmermann M, Ito Y, Hess S, Fujieda K, Molinari L (2005). High thyroid volume in children with excess dietary iodine intakes. Am J Clin Nutr.

[B33] Delange F, de Benoist B, Alnwick D (1999). Risks of iodine-induced hyperthyroidism after correction of iodine deficiency by iodized salt. Thyroid.

[B34] WHO/ICCIDD/UNICEF (Ed.) (1996). Recommended iodine levels in salt and guidelines for monitoring adequacy and effectiveness Volume WHO/NUT/9613.

[B35] Eckhoff K, Maage A (1997). Iodine Content in Fish and Other Food Products from East Africa Analyzed by ICP-MS. Journal of Food Composition and Analysis.

[B36] Sharp P, Geissler C, Powers H (2005). Minerals and trace elements: iodine. Human Nutrition.

[B37] UNICEF (2008). Protecting children's brain development through universal salt iodization: Successes and Challenges in Eastern and Southern Africa.

[B38] ICCIDD, MI (2007). Supporting small scale salt producers is essential for achieving USI. IDD Newsletter.

[B39] Pearce EN, Gerber AR, Gootnick DB, Khan LK, Li R, Pino S, Braverman LE (2002). Effects of chronic iodine excess in a cohort of long-term American workers in West Africa. J Clin Endocrinol Metab.

[B40] Todd CH, Allain T, Gomo ZA, Hasler JA, Ndiweni M, Oken E (1995). Increase in thyrotoxicosis associated with iodine supplements in Zimbabwe. Lancet.

[B41] WHO/ICCIDD/UNICEF (1997). Review of findings from seven-country study in Africa on levels of salt iodization in relation to iodine deficiency disorders including iodine induced hyperthyroidism.

[B42] Okosieme OE (2006). Impact of iodination on thyroid pathology in Africa. J R Soc Med.

[B43] Ategbo E, Sankar R, Schultink W, Haar F van der, Pandav C (2008). An assessment of progress towards universal salt iodization in Rajasthan, India, using iodine nutrition indicators in school-aged children and pregnant women from the same households. Asia Pacific J Clinical Nutrition.

[B44] Sundqvist J, Wijetunga M, Assey V, Gebre-Medhin M, Peterson S (1998). Salt iodation and risk of neonatal brain damage. Lancet.

[B45] Sullivan K, Suchdev P, Grummer-Strawn L (2008). Achieving and Sustaining USI: Doing It Well Through Quality Assurance. SCN News.

[B46] Andersson M, Berg G, Eggertsen R, Filipsson H, Gramatkovski E, Hansson M, Hulthén L, Milakovic M, Nyström E (2008). A new national study suggests iodine intakes are adequate in Sweden. IDD Newsletter.

[B47] Torheim L, Granli G, Sidibe C, Traore A, Oshaug A (2004). Women's iodine status and its determinants in an iodine-deficient area in the Kayes region, Mali. Public Health Nutrition.

