# Supplementary material for: Tanzania national survey on iodine deficiency: impact after twelve years of salt iodation
Source: BMC Public Health. 2009 Sep 3;9:319. doi: 10.1186/1471-2458-9-319 (PMC2749826; doi:10.1186/1471-2458-9-319)
Supplement: Additional file 1 — Proportion of use of iodated salt (I-salt)* at household level and TGP** at regional level and median UIC*** at district level in schoolchildren (6 – 12 years) in 2004 ordered according to regional I-salt coverage. [file 1471-2458-9-319-S1.doc]

**Additional file 1
File format**: DOC
**Title**: Proportion of use of iodated salt (I-salt)* at household level and TGP** at regional level and median UIC*** at district level in schoolchildren (6 – 12 years) in 2004 ordered according to regional I-salt coverage 
**Description**: The data provided represent the statistical analysis of I-salt consumption at households, prevalence of goitre and status of median UIC levels in school children.

| **Results by region** | | | | | | | | | **Results for the district in each region that was selected for urinary iodine concentration measurement** | | | | | | | | | |
| --- | --- | --- | --- | --- | --- | --- | --- | --- | --- | --- | --- | --- | --- | --- | --- | --- | --- | --- |
| Region | Districts  per region | Household use of iodated salt | | | Total goitre prevalence | | | | Household use of iodated salt | | | | Total goitre prevalence | | Urinary iodine concentrations | | | |
|  |  | Number of salt samples tested | Samples with iodine n (%) | Traffic light status | Total number of children examined | Children with goitre n (%) | Traffic light status | District | Number of salt samples tested | Samples with iodine n (%) | Traffic light status | Total number of children examined | Children with goitre n (%) | Traffic light status | Number of urine samples analysed | Median (µg/L) | Traffic light status | % children with UIC  ≥300 µg/L |
| Kagera | 5 | 6739 | 6716 (99.7) | G | 4574 | 35 ( 0.8) | G | Bukoba | 816 | 816 ( 100.0) | G | 549 | 6 ( 1.1) | G | 125 | 165.5 | G | 18.4 |
| Kigoma | 3 | 6937 | 6909 (99.6) | G | 4516 | 109 ( 2.4) | G | Kibondo | 1426 | 1426 (100.0) | G | 815 | 41 ( 5.0) | Y | 154 | 254.6 | G | 37.0 |
| Mara | 4 | 5717 | 5696 (99.6) | G | 4254 | 623 (14.4) | Y | Musoma | 1521 | 1514 ( 99.5) | G | 1139 | 174 (15.3) | Y | 131 | 230.5 | G | 33.6 |
| Mwanza | 8 | 12497 | 12438 (99.5) | G | 8509 | 24 ( 0.3) | G | Ilemela | 1412 | 1406 ( 99.6) | G | 995 | 0 ( 0.0) | G | 107 | 326.5 | P | 56.1 |
| Tabora | 6 | 7985 | 7881 (98.7) | G | 5966 | 116 ( 1.9) | G | Sikonge | 926 | 926 ( 100.0) | G | 701 | 1 ( 0.1) | G | 121 | 234.8 | G | 38.0 |
| Arusha | 5 | 8658 | 8549 (98.7) | G | 6408 | 576 ( 9.0) | Y | Monduli | 1075 | 1063 ( 98.9) | G | 737 | 120 (16.3) | Y | 116 | 275.5 | G | 47.4 |
| Morogoro | 5 | 5544 | 5306 (95.7) | G | 4773 | 223 ( 4.7) | G | Kilosa | 917 | 896 ( 97.7) | G | 808 | 31 ( 3.8) | G | 117 | 182.1 | G | 18.8 |
| Dodoma | 5 | 6647 | 6292 (94.7) | G | 4349 | 166 ( 3.8) | G | Dodoma | 2293 | 2283 ( 99.6) | G | 1895 | 70 ( 3.7) | G | 105 | 215.6 | G | 30.5 |
| Mbeya | 7 | 7534 | 6640 (88.1) | Y | 5628 | 956 (17.0) | Y | Mbeya | 1066 | 1055 ( 99.0) | G | 691 | 135 (19.5) | Y | 107 | 134.4 | G | 6.5 |
| Shinyanga | 7 | 9160 | 8005 (87.4) | Y | 5855 | 66 ( 1.1) | G | Kishapu | 1036 | 939 ( 90.6) | G | 665 | 3 ( 0.4) | G | 119 | 215.5 | G | 26.1 |
| Dar es Salaam | 3 | 2420 | 2094 (86.5) | Y | 2631 | 8 ( 0.3) | G | Temeke | 937 | 663 ( 70.8) | Y | 993 | 2 ( 0.2) | G | 130 | 936.7 | P | 88.5 |
| Ruvuma | 4 | 4175 | 3523 (84.4) | Y | 2681 | 46 ( 1.7) | G | Namtumbo | 558 | 494 ( 88.5) | Y | 259 | 1 ( 0.4) | G | 85 | 42.4 | R | 1.2 |
| Singida | 3 | 4172 | 3521 (84.4) | Y | 2358 | 56 ( 2.4) | G | Singida | 1180 | 791 ( 67.0) | Y | 595 | 12 ( 2.0) | G | 101 | 91.6 | Y | 10.9 |
| Kilimanjaro | 5 | 5927 | 4856 (81.9) | Y | 4607 | 184 ( 4.0) | G | Hai | 1215 | 1215 (100.0) | G | 986 | 31 ( 3.1) | G | 152 | 399.0 | P | 65.1 |
| Pwani | 6 | 4791 | 3730 (77.9) | Y | 3396 | 10 ( 0.3) | G | Kisarawe | 1107 | 971 ( 87.7) | Y | 763 | 0 ( 0.0) | G | 114 | 813.0 | P | 78.9 |
| Tanga | 7 | 6125 | 4627 (75.5) | Y | 5555 | 215 ( 3.9) | G | Muheza | 899 | 485 ( 53.9) | Y | 734 | 32 ( 4.4) | G | 208 | 187.3 | G | 32.7 |
| Manyara | 5 | 7047 | 5267 (74.7) | Y | 4866 | 541 (11.1) | Y | Simanjiro | 1154 | 1154 (100.0) | G | 889 | 66 ( 7.4) | Y | 138 | 412.3 | P | 72.5 |
| Mtwara | 4 | 5237 | 3368 (64.3) | Y | 3645 | 1 ( 0.0) | G | Masasi | 1967 | 1326 ( 67.4) | Y | 1417 | 0 ( 0.0) | G | 113 | 57.6 | Y | 6.2 |
| Rukwa | 3 | 3797 | 1414 (37.2) | R | 2610 | 367 (14.1) | Y | Mpanda | 1186 | 729 ( 61.5) | Y | 881 | 110 (12.5) | Y | 120 | 262.1 | G | 45.8 |
| Iringa | 6 | 6711 | 2471 (36.8) | R | 4275 | 838 ( 19.6) | Y | Mufindi | 1052 | 238 ( 22.6) | R | 702 | 110 (15.7) | Y | 129 | 88.0 | Y | 2.3 |
| Lindi | 5 | 4121 | 1048 (25.4) | R | 2590 | 21 ( 0.9) | G | Liwale | 534 | 307 ( 57.5) | Y | 285 | 2 ( 0.7) | G | 148 | 63.3 | Y | 0.0 |
| Total/unweighted mean | **106** | **131941** | **110350 (83.6)** | **Y** | **94046** | **5181 (5.5)** | **Y** | **Total** | **24277** | **20697 (85.3)** | **Y** | **17499** | **947 ( 5.4)** | **Y** | **2640** | **203.3** | **G** | **35.1** |

NB: Overall coverage (national) for iodated salt* = 83.6% (95% CI: 83.4, 83.8), Total goitre prevalence** = 5.5% (95%CI: 5.3, 5.6),

Overall I-salt coverage for districts sub-sampled for urinary iodine concentration*** = 85.3% (95% CI: 84.9, 85.8), TGP = 5.4 % (95 % CI 5.3, 5.5). Overall median UIC = 203.3 (95% CI: 187, 219) µg/L

Key to traffic light alphabetical colour codes (according to WHO [1] except for iodated salt, where two more categories were added):

- I-salt coverage: 0 - 49.9% (very poor) = red (R), 50 - 90% (poor/unsatisfactory) = yellow (Y), >90% (adequate) = green (G)
- TGP: 0 - 4.9 % (not of public health significance) = green (G), 5 - 19.9% (mild) = yellow (Y), 20 - 29.9% (moderate) = orange (O), ≥30% (severe) = red (R)
- Median urinary iodine: 0 - 49.9 µg/L (very insufficient) = red (R), 50-99.9 µg/L (insufficient) = yellow (Y), 100 - 299.9 µg/L optimal and above requirement = green (G), ≥300 µg/L (excessive intake) = purple (P).
