# Supplementary material for: Tanzania national survey on iodine deficiency: impact after twelve years of salt iodation
Source: BMC Public Health. 2009 Sep 3;9:319. doi: 10.1186/1471-2458-9-319 (PMC2749826; doi:10.1186/1471-2458-9-319)
Supplement: Additional file 2 — Proportion of use of iodated salt (I-salt)* at household level, TGP** at regional level and median UIC*** at district level in schoolchildren (6 – 18 years) in 2004 ordered according to regional I-salt coverage. [file 1471-2458-9-319-S2.doc]

**Additional file 2
File format**: DOC
**Title**: Proportion of use of iodated salt (I-salt)* at household level and TGP** at regional level and median UIC*** at district level in schoolchildren (6 – 18 years) in 2004 ordered according to regional I-salt coverage
**Description**: The data provided represent the statistical analysis of I-salt consumption at households, prevalence of goitre and median UIC levels in all school children age-group 6-18 years.

| **Results by region** | | | | | | | | | **Results for the district in each region that was selected for urinary iodine concentration measurement** | | | | | | | | | | |
| --- | --- | --- | --- | --- | --- | --- | --- | --- | --- | --- | --- | --- | --- | --- | --- | --- | --- | --- | --- |
| Region | Districts  per region | Household use of iodated salt | | | Total goitre prevalence | | | | Household use of iodated salt | | | | Total goitre prevalence | | | Urinary iodine concentrations | | | |
|  |  | Number of salt samples tested | Samples with iodine n (%) | Traffic light status | | Total number of children examined | Children with goitre n (%) | Traffic light status | District | Number of salt samples tested | Samples with iodine n (%) | Traffic light status | Total number of children examined | Children with goitre n (%) | Traffic light status | Number of urine samples analysed | Median (µg/L) | Traffic light status | % children with UIC ≥300 µg/L |
| Kagera | 5 | 6739 | 6716 (99.7) | G | | 7147 | 118 ( 1.7) | G | Bukoba | 816 | 816 ( 100.0) | G | 842 | 16 ( 1.9) | G | 209 | 192.5 | G | 26.8 |
| Kigoma | 3 | 6937 | 6909 (99.6) | G | | 7037 | 437 ( 6.2) | Y | Kibondo | 1426 | 1426 ( 100.0) | G | 1426 | 186 (13.0) | Y | 210 | 254.6 | G | 38.1 |
| Mara | 4 | 5717 | 5696 (99.6) | G | | 6038 | 968 (16.0) | Y | Musoma | 1521 | 1514 ( 99.5) | G | 1677 | 285 (17.0) | Y | 211 | 230.5 | G | 32.2 |
| Mwanza | 8 | 12497 | 12438 (99.5) | G | | 12635 | 122 ( 1.0) | G | Ilemela | 1412 | 1406 ( 99.6) | G | 1429 | 1 ( 0.10 | G | 209 | 372.5 | P | 59.8 |
| Tabora | 6 | 7985 | 7881 (98.7) | G | | 9251 | 229 ( 2.5) | G | Sikonge | 926 | 926 ( 100.0) | G | 1094 | 4 ( 0.4) | G | 210 | 236.4 | G | 36.7 |
| Arusha | 5 | 8658 | 8549 (98.7) | G | | 8659 | 851 ( 9.8) | Y | Monduli | 1075 | 1063 ( 98.9) | G | 1075 | 183 (17.0) | Y | 209 | 280.0 | G | 48.3 |
| Morogoro | 5 | 5544 | 5306 (95.7) | G | | 6437 | 343 ( 5.3) | Y | Kilosa | 917 | 896 ( 97.7) | G | 1167 | 51 ( 4.4) | G | 212 | 166.7 | G | 20.8 |
| Dodoma | 5 | 6647 | 6292 (94.7) | G | | 6562 | 314 ( 4.8) | G | Dodoma | 2293 | 2283 ( 99.6) | G | 2105 | 113 ( 5.4) | G | 210 | 203.6 | G | 28.1 |
| Mbeya | 7 | 7534 | 6640 (88.1) | Y | | 8957 | 1754 (19.6) | Y | Mbeya | 1066 | 1055 ( 99.0) | G | 1121 | 271 (24.2) | O | 208 | 147.0 | G | 9.1 |
| Shinyanga | 7 | 9160 | 8005 (87.4) | Y | | 9160 | 105 ( 1.1) | G | Kishapu | 1036 | 939 ( 90.6) | G | 1036 | 11( 1.1) | G | 208 | 224.5 | G | 32.7 |
| Dar es Salaam | 3 | 2420 | 2094 (86.5) | Y | | 3438 | 9 ( 0.3) | G | Temeke | 937 | 663 ( 70.8) | Y | 1329 | 2 ( 0.2) | G | 211 | 887.0 | P | 87.2 |
| Ruvuma | 4 | 4175 | 3523 (84.4) | Y | | 4190 | 339 ( 8.1) | Y | Namtumbo | 558 | 494 ( 88.5) | Y | 559 | 60 ( 10.7) | G | 206 | 45.1 | R | 1.5 |
| Singida | 3 | 4172 | 3521 (84.4) | Y | | 4176 | 88 ( 2.1) | G | Singida | 1180 | 791 ( 67.0) | Y | 1183 | 27 ( 2.3) | G | 210 | 90.2 | Y | 10.0 |
| Kilimanjaro | 5 | 5927 | 4856 (81.9) | Y | | 5928 | 338 ( 5.7) | Y | Hai | 1215 | 1215 ( 100.0) | G | 1215 | 42 ( 3.5) | G | 220 | 396.5 | P | 65.5 |
| Pwani | 6 | 4791 | 3730 (77.9) | Y | | 5310 | 28 ( 0.5) | G | Kisarawe | 1107 | 971 ( 87.7) | Y | 1207 | 1 ( 0.1) | G | 210 | 836.3 | P | 78.1 |
| Tanga | 7 | 6125 | 4627 (75.5) | Y | | 7783 | 301 ( 3.9) | G | Muheza | 899 | 485 ( 53.9) | Y | 1052 | 45 ( 4.3) | G | 325 | 185.7 | G | 31.4 |
| Manyara | 5 | 7047 | 5267 (74.7) | Y | | 7048 | 729 (10.3) | Y | Simanjiro | 1154 | 1154 ( 100.0) | G | 1154 | 75 ( 6.5) | Y | 211 | 412.4 | P | 72.5 |
| Mtwara | 4 | 5237 | 3368 (64.3) | Y | | 5240 | 1 ( 0.0) | G | Masasi | 1967 | 1326 ( 67.4) | Y | 1968 | 0 ( 0.0) | G | 212 | 64.7 | Y | 10.8 |
| Rukwa | 3 | 3797 | 1414 (37.2) | R | | 4603 | 971 (21.1) | O | Mpanda | 1186 | 729 ( 61.5) | Y | 1453 | 260 (17.9) | Y | 201 | 270.8 | G | 47.8 |
| Iringa | 6 | 6711 | 2471 (36.8) | R | | 6845 | 1694 (24.7) | Y | Mufindi | 1052 | 238 ( 22.6) | R | 1054 | 330 (31.3) | R | 212 | 92.9 | Y | 3.3 |
| Lindi | 5 | 4121 | 1048 (25.4) | R | | 4314 | 30 ( 0.7) | G | Liwale | 534 | 307 ( 57.5) | Y | 564 | 4 ( 0.7) | G | 209 | 72.6 | Y | 0.0 |
| Total/unweighted mean | **106** | **131941** | **110350 (83.6)** | **Y** | | **140758** | **9769 (6.9)** | **Y** | **Total** | **24277** | **20697 ( 85.3)** | **Y** | **25710** | **1980 ( 7.7)** | **Y** | **4523** | **203.6** | G | 35.2 |

NB: Overall coverage (national) of iodated salt* = 83.6% (95% CI: 83.4, 83.8), Total goitre prevalence** = 6.9% (95%CI: 6.8 - 7.1),

Overall I-salt coverage for districts sub-sampled for UIC *** = 85.3% (95% CI: 84.9, 85.8), TGP = 7.7 % (95 % CI: 7.6 - 7.8). Overall median UIC = 203.6 (95% CI: 192.0, 215.2) µg/L

Key to traffic light alphabetical colour codes (according to WHO [1] except for iodated salt, where two more categories were added):

- I_salt coverage: 0 - 49.9% (very poor) = red (R), 50 - 90% (poor/unsatisfactory) = yellow (Y), >90% (adequate) = green (G)
- TGP: 0 - 4.9 % (not of public health significance) =green (G), 5 - 19.9% (mild) = yellow (Y), 20 - 29.9% (moderate) = orange (O), ≥30% (severe) = red (R)
- Median urinary iodine: 0 - 49.9 µg/L (very insufficient) = red (R), 50-99.9 µg/L (insufficient) = yellow (Y), 100 - 299.9 µg/L optimal and above requirement= green (G),

≥300 µg/L (excessive intake) = purple (P).
